# Antiviral activity of galvanic microcells of zinc and copper contained within painted surfaces

**DOI:** 10.1038/s41598-022-05330-8

**Published:** 2022-01-25

**Authors:** Wojciech Spisak, Mariusz Kaszczyszyn, Mateusz Szar, Jarosław Kozak, Krzysztof Stachowicz

**Affiliations:** 1 Research & Development Centre ALCOR Ltd., Kępska 12, 45-130 Opole, Poland; 2 W. Szafer Institute of Botany, Polish Academy of Sciences, Lubicz 46, 31-512 Cracow, Poland

**Keywords:** Chemical engineering, Chemical physics, Microbiology

## Abstract

This study presents research related to the antiviral activity of painted surfaces containing galvanic microcells of zinc and copper. The aim of this study was to investigate the virucidal activity of galvanic microcells of zinc and copper grains fixed with adequate homogeneity and degree of aggregation in water-based acrylic paint layers in reference to a non-treated reference control. This paper provides evidence that a paint coating with a total copper surface area of 4.4% displays antiviral activity against human coronavirus NL63 according to ISO 21702 and inactivates > 99% of virus after 4 h of contact relative to a non-treated reference control.

## Introduction

The most common way people become infected with viruses is through exposure to respiratory droplets. In addition, virus-laden droplets can also be deposited on surfaces, forming fomites, which serve as a secondary source of transmission when touched^1^. It has been reported that SARS-CoV-2 can survive on plastic for 72 h, on stainless steel for 48 h, on cardboard for 24 h, and on copper for 4 h^2^.

The WHO recommended surface disinfection to reduce the risk of contamination from the fomite route. However, excessive use of disinfectants poses a potential threat to human health^3^. Previous literature studies confirm a link between exposure to cleaning agents and disinfectants and adverse respiratory effects, exacerbation of asthma and new onset asthma in cleaning personnel, private homes and healthcare professionals^4,5^. Surface disinfection is one of the most recommended methods to reduce the risk of transmission of viruses, including SARS-CoV-2. Since the beginning of 2020, there has been an increasing number of reports of acute health effects due to misuse and overexposure to disinfectants^6^.

Therefore, it is necessary to develop new methods for ensuring continuous antiviral protection of surfaces to eliminate viruses shortly after contamination.

We hypothesized that paint coatings containing galvanic microcells of zinc and copper with adequate homogeneity and degree of aggregation would shorten the infectivity of deposited viruses on the treated surfaces.

All metals can be classified into a galvanic series representing the electrical potential against a standard reference electrode usually Standard Hydrogen Electrode. When two or more dissimilar metals come into contact in an electrolyte galvanic cells is created. One metal with lover electrical potential acts as anode and the other as cathode. The electropotential difference between these two electrodes is the driving force for the ions forming galvanic current^7^. We use name galvanic microcells when metal electrodes are in form of grains within equivalent diameter less than 100 µm.

Zinc and copper are well-known antifungal and antibacterial metals. Copper was registered by the United States Environmental Protection Agency in 2008 as the first antimicrobial solid surface material. Extensive research has confirmed that copper surfaces kill 99.9% of microbes after a contact time of 2 h^8^. According to recent research SARS-CoV-1 and SARS-CoV-2 become inactivated on pure copper surfaces within 4 h^2^. Reduction in Murine Norovirus following 2-h exposure to the paint containing copper-glass ceramic particles was ≥ 99.97%^9^.

Copper oxide impregnated countertops demonstrated over a 3 log (> 99.9%) reduction against bacterial strains *Staphylococcus aureus*, *Enterobacter aerogenes*, *Pseudomonas aeruginosa*, *Methicillin* resistant *Staphylococcus aureus* and *Escherichia coli* as compared to the control countertops without copper^10^.

It has been confirmed in our previous research that galvanic microcells of zinc and copper show antifungal efficiency by creating electrochemically activated, ionically conductive conditions^11^. The tests were also carried out in accordance with the standard EN 15457^12^. Samples containing galvanic microcells of zinc and copper showed stable effectiveness (level 0 according to standard) against the tested fungal strains: *Cladosporium cladosporioides*, *Ulocladium atrum*, *Aspergillus versicolor*, *Aspergillus Niger* in the maximum time of tests carried out in accordance with the standard, i.e. after 21 days, while tested in the same time and conditions, the control samples showed full viability and growth of the test strains (level 4 ) after only 7 days^13^. The addition of a third bismuth electrode also results in the system efficiency against *Micrococcus luteus* bacterial strain^14^.

In our current study it was investigated whether a paint coating containing galvanic microcells of zinc and copper with a total copper surface area of 4.4% demonstrate virucidal activity in reference to a non-treated reference control.

## Materials and methods

### Galvanic microcells

Electrodes in the form of metal grains were used in the experiment. The zinc powder, with a minimum content of 99.9% zinc, was formed by grinding solid Zn material in a ball mill. The ground zinc powder had a grain size of less than 100 μm (prod. Libra Sp. z o.o., Trzebinia, Poland). The metallic copper had a content of 99.5–99.7% copper and was obtained in the form of a powder precipitated from a copper sulfate solution. The grain size was less than 100 μm and was obtained as a result of cathodic deposition during electrolysis of aqueous copper sulfate solutions (prod. Libra Sp. z o.o.,Trzebinia, Poland).

### Water-based acrylic paint

White water-based acrylic paint, meant for interior application, with a density 1.38–1.4 g/cm^3^, a solids content by weight of 50.5–52.5%, a VOC (volatile organic compounds) < 30 g/dm^3^, a pH of 8–9, and a dynamic viscosity room temperature) of 5000 to 6000 mPa·s, (prod. Martin Mathys N.V., Zelem, Belgium ) was used for the experiment.

### Finite element model (FEM) of the electric field

Modelling electromagnetic fields as a result of galvanic action was based on the finite element model (FEM). The FEM package used to create the electric field models was Wolfram Mathematica R10 Multiphysics (https://www.wolframalpha.com). The standard electrode potential was − 0.7628 V for zinc and + 0.337 V for copper relative to the standard hydrogen electrode, and spherically shaped electrodes were used in the geometric electric field calculations. The calculation field was 300 μm × 300 μm. Electrodes with diameters of 15 μm were used. Electric field voltages were modelled for electrode distances of 250, 200, 150, 100, 75, and 50 μm.

### Assessment of the homogeneity of the distribution of galvanic microcells in the coating using a probabilistic method

To verify the effectiveness of copper in the tested coating, it is necessary to determine the homogeneity of its distribution in the dry film. The homogeneity of the galvanic microcells distribution in the paint layer was determined and assessed using a probabilistic method with a mathematical model of three circles. This method is also used in astronomy and cosmology for describing the population distribution of galaxies^15,16^. Recently, a similar image analysis method, originally used in astronomy, was applied toward the pathologic analysis of specimens from patients with skin cancer^17^.

In this paper, we present an attempt to formulate a quantitative criterion for determining the homogenous and heterogeneous patterns of copper cathode distribution within a water-based coating based on probabilistic analysis. We propose a way of calculating a coefficient of copper cathode homogeneity in water-based paint based only on measurable geometric relations. The coefficient could be a quantitative criterion for the evaluation of copper cathode homogeneity. The discussed homogeneity coefficient that is determined according to geometric relations may be useful in the investigation of the antiviral activity of painted surfaces. From the analysis of microscopic photographs showing galvanic microcells of zinc and copper within the painted surface, we investigated whether there were any special properties of the galvanic microcell distribution in the water-based paint and how this distribution should be described.

**Figure 1:**
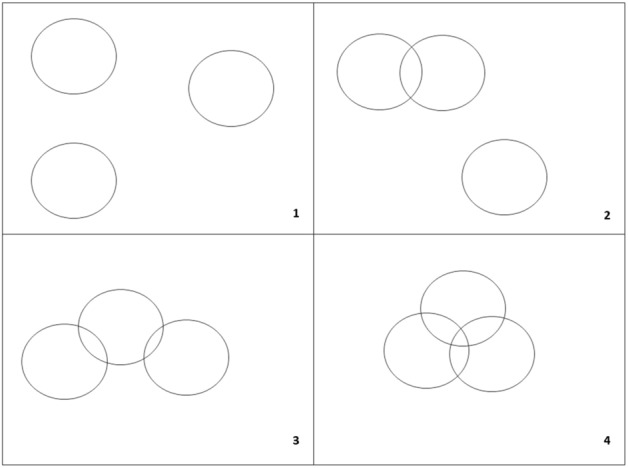
Possible classes of the configuration of three circles. Source: Gancarek et al.^16^.

Description of the method: Taking a two-dimensional metric surface with points distributed within it as an example. C(x,r) is a circle (circular surroundings of point x) with radius r. All possible sets consisting of three circles C(x,r) chosen from a given n-element population can be classified into 4 divisions^15^ (Fig. 1):

1. All three circles are disjointed.
2. Two circles have nonempty sections, and the third circle is disjointed with each of them.
3. One circle has a nonempty section with the others, which are disjointed.
4. Each circle has a nonempty section with each of the others.

For a given *r, the* frequency of occurrence of each of the classes $l_{i} \left( r \right){ }$ (i = 1, 2, 3, 4) may be calculated and compared with the theoretical frequency $l_{i} \left( r \right)$ for a random distribution, i.e., the arrangement where each position for each of three points is possible to the same degree.

To determine the differences between the investigated microelectrode distribution and the random distribution, the factor $z_{i} \left( r \right)$ is introduced:

$$z_{i} \left( r \right) = \frac{{l_{i} \left( r \right){ }}}{{\overline{{l_{i} \left( r \right)}} }}$$

The frequency of occurrence of the sequence of classes presented in Fig. 1$\overline{{l_{i} \left( r \right)}}$ is as follows:

$$\begin{aligned} {\text{Class 1}}:\overline{{l_{1} \left( r \right)}} & = S^{ - 2} (S^{2} - 12\pi r^{2} S + 32\pi^{2} r^{4} + 12\sqrt 3 \pi r^{4} \\ {\text{Class 2}}:\overline{{l_{2} \left( r \right)}} & = 12\pi r^{2} S^{ - 2} \left[ {S - r^{2} \left( {4\pi + 3\sqrt 3 } \right)} \right] \\ {\text{Class 3}}:\overline{{l_{3} \left( r \right)}} & = 36\sqrt 3 \pi r^{4} S^{ - 2} \\ {\text{Class 4}}:\overline{{l_{4} \left( r \right)}} & = 4\pi r^{4} S^{ - 2} \left( {4\pi - 3\sqrt 3 } \right) \\ \end{aligned}$$

$l_{i} \left( r \right)$, frequency of occurrence of each class; $\overline{{l_{i} \left( r \right)}}$, theoretical frequency of occurrence of each class; r, radius [µm]; S, area [µm^2^]; $z_{1} \left( r \right)$, measure of scattering; $z_{2} \left( r \right)$, measure of weak compactness; $z_{3} \left( r \right)$, measure of compactness; $z_{4} \left( r \right)$, measure of strong compactness.

### Testing the virucidal activity of galvanic microcells of zinc and copper within painted surfaces

Antiviral test procedure according to ISO 21702:2019^18^ was performed by Virology Research Services ltd, London. Treated and non-treated materials were placed in individual discs in triplicate. A liquid volume was 200 μl of an appropriate concentration of virus (1 × 10^5 PFU/ml stock of human coronavirus NL63) and was added onto each surface and covered with an inert film. A lid was placed over each disc, which was then incubated for 4 h at room temperature in a humidified chamber. At the end of the incubation, the film was lifted, and the sample was washed with media to recover the virus. The amount of infectious virus recovered from each sample was then quantified by TCID50.

As a further control, virus was added to three pieces of the reference control material and immediately recovered by washing (referred to as the ‘virus recovery control’ or ‘back-titration’). This recovered virus was used to quantify the starting amount of virus.

TCID50 determination A seven-point, ten-fold serial dilution from the virus-containing wash media was tested in quadruplicate for each sample on Rhesus Monkey Kidney Epithelial (LLC-MK2) cells. After 10 days, a viability crystal violet assay was carried out to determine cell viability across the dilution series. The dilution at which 50% of cells are infected/killed (TCID50) was calculated using a regression analysis. Quantification of antiviral activity was calculated as follows: R = Ut-At.

where Ut is the average of the common logarithm of the number of infectious units recovered from the untreated test specimens at the end of the incubation time; and At is the average of the common logarithm of the number of infectious units recovered from the treated test specimens at the end of the incubation time. An R value of ≥ 1 indicates antiviral activity.

Glass samples for tests were cut according to ISO 21702:2019. The samples were cut into 50 mm × 50 mm squares with a thickness of < 10 mm. The glass samples were covered with acrylic paint containing galvanic microcells. As a reference control glass samples not treated with acrylic paint containing galvanic microcells were used.

Human coronavirus NL63 and Host cells of Rhesus Monkey Kidney Epithelial (LLC-MK2) were used for the tests. Test inoculum volume was 200 μl. Virus titre was 1 × 10^5. In our research contact time of 4 h was investigated.

### Photography and microelectrode counting methods

**Figure 2:**
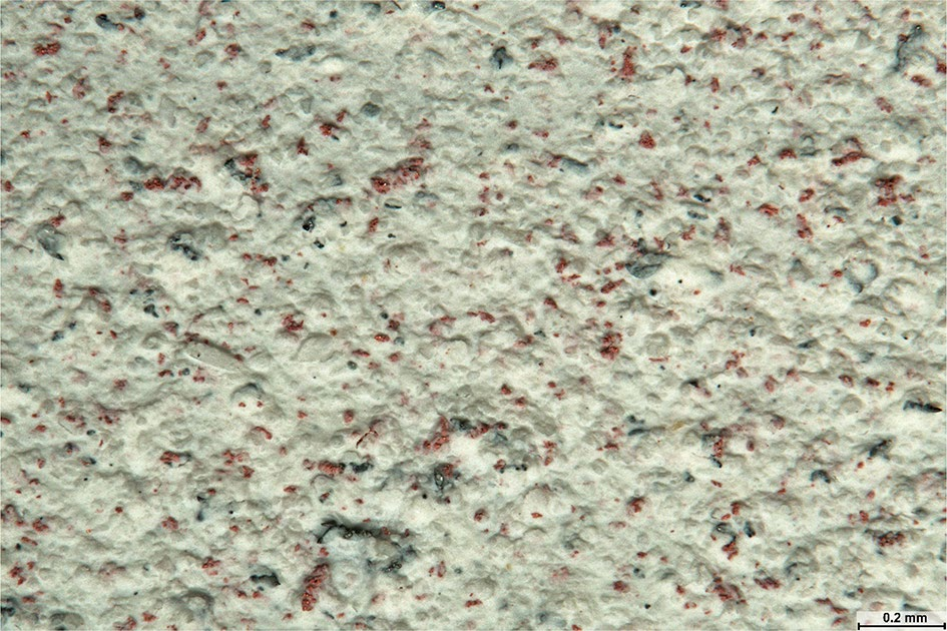
Microscopic image of copper cathode and zinc anode distribution in water based coating with side lighting.

**Figure 3:**
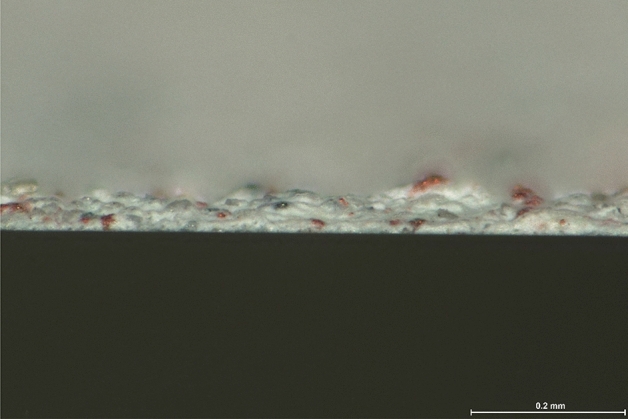
Cross section of the acrylic paint coating with galvanic microcells surface structure.

Photographs of galvanic microcell electrodes in the acrylic paint coating were taken with focus stacking using a Leica M 205 stereomicroscope, including a Leica DMC 5400 20-Mpx camera, as well as Las X and Helicon Focus 7.6.4 Pro software. Randomly selected rectangular 3.2 mm^2^ and 0.4 mm^2^ sample areas were photographed with uniform, shadow-free illumination and at 60 × and 161 × magnification. The count of all visible microelectrodes was determined with SmartGrain image analysis software^19^. For maximum counting accuracy, the colour saturation of the photographed metal grains was increased by 60% with Adobe Photoshop Elements 15.0 software. The microelectrode location coordinates, used to evaluate the uniformity of distribution, were determined by a qualified observer with Las X image acquisition software. Images showing the surface structure of the acrylic paint coating were taken from the side, the top (Fig. 2), and of the cross-section (Fig. 3).

## Results and discussion

**Figure 4:**
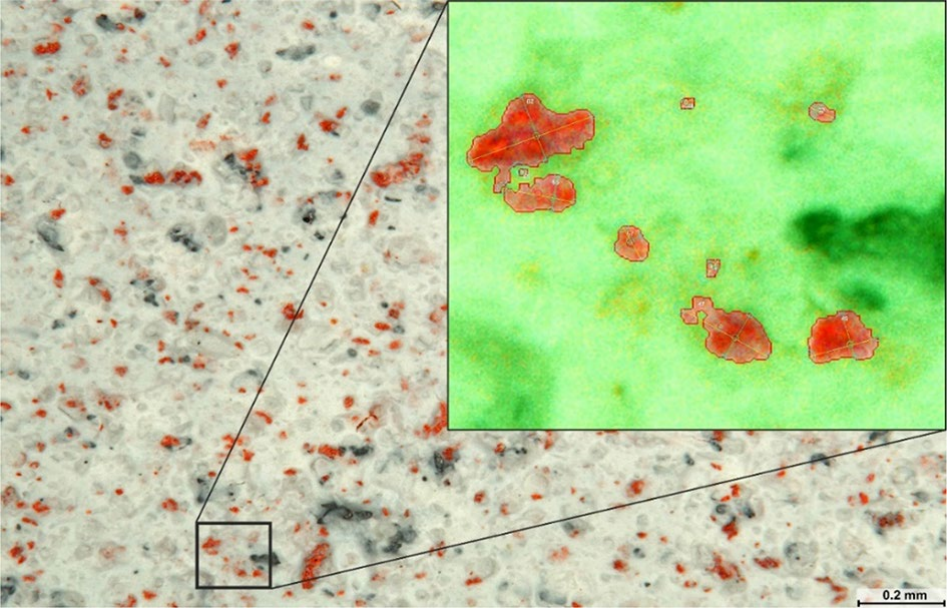
Number and size of copper grains marked in red contrast.

Copper cathodes were taken into account in our research on the homogeneity of the grain distribution. The copper cathode distribution in the coating was assessed based on a probabilistic method. Briefly, 715 points (copper grains) were identified in a microscopic image (60 × magnification) of a coating containing galvanic microcells of zinc and copper applied on a solid surface; these were used for illustrating the grain distribution in the coating. The count of all visible microelectrodes was determined with SmartGrain image analysis software with the colour saturation increased by 60% (Fig. 4).

**Figure 5:**
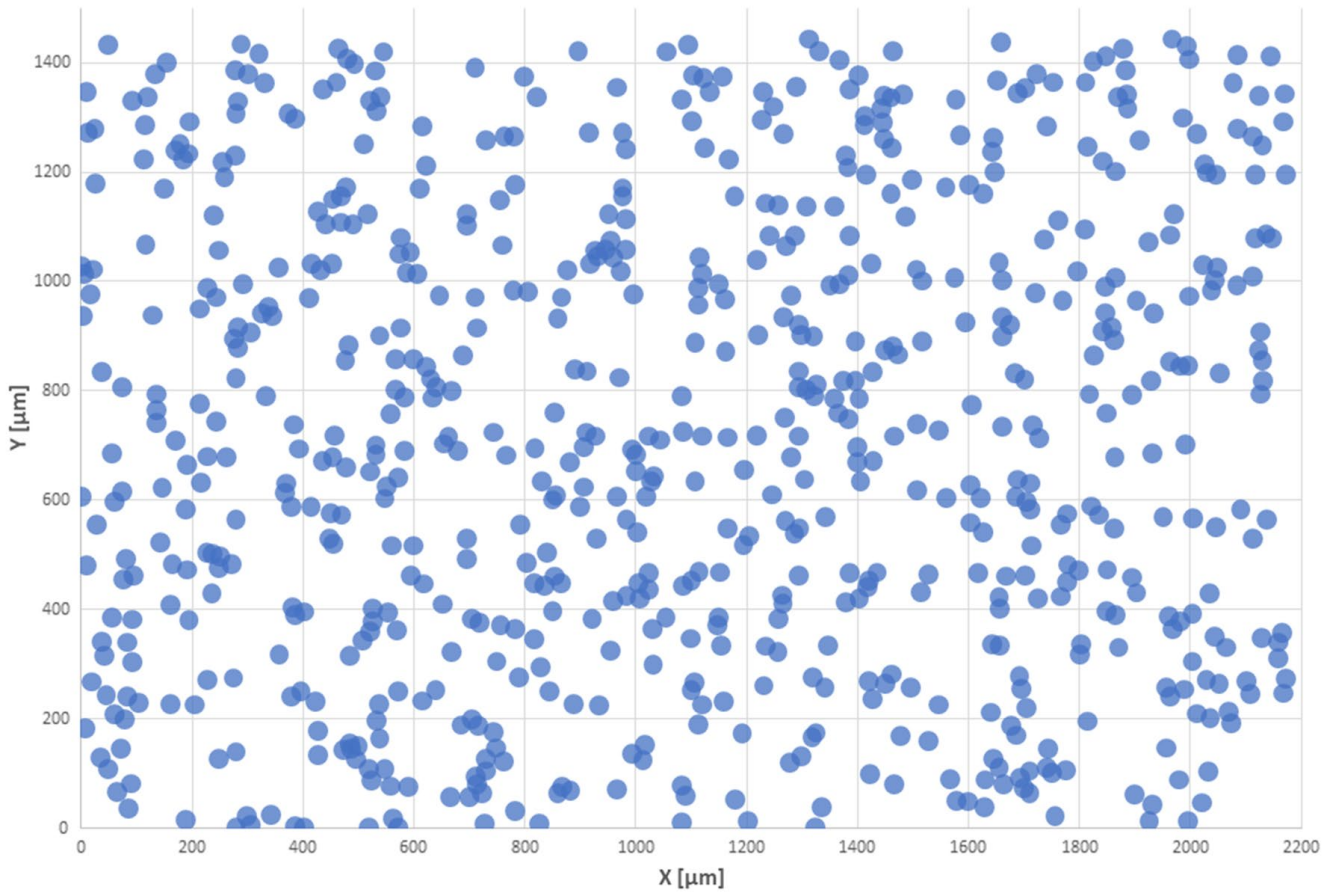
Analysed distribution of points for r = 15 μm. The area of the analysed surface was 2174 μm × 1447 μm.

The coordinates of the points were marked on the X and Y axes (Fig. 5).

Collecting the above data allowed for the evaluation of the copper cathode distribution in the coating based on a probabilistic method (see the Methods). Calculations of the distribution of grains on the surface are shown in Table 1. Index $z_{i} \left( r \right)$ is a measure of the dissipation of objects and $z_{1} \left( r \right),$ $z_{2} \left( r \right)$, $z_{3} \left( r \right)$, $z_{4} \left( r \right)$ are measures of the concentrations in the population.

**Table 1:** Calculations of the distribution of grains on the surface in relation to the random distribution for $\left( r \right)$ of 5, 10, 15, 20, 25, 50, and 100 μm.

r	i	1	2	3	4
5	n_i_(r)	60,665,605	0	0	0
	v_i_(r)	1	0	0	0
	P_i_(r)	0,999,700,423	0,000,299,559	1,23719E−08	5,84944E−09
	z_i_(r)	1,000,299,667	0	0	0
10	n_i_(r)	60,601,459	64,125	18	3
	v_i_(r)	0,99,894,263	0,001,057,024	2,96708E−07	4,94514E−08
	P_i_(r)	0,998,801,982	0,001,197,727	1,97951E−07	9,3591E−08
	z_i_(r)	1,000,140,817	0,882,525,147	1,498,901,565	0,528,377,912
15	n_i_(r)	60,472,556	192,911	102	36
	v_i_(r)	0,996,817,818	0,003,179,907	1,68135E−06	5,93417E−07
	P_i_(r)	0,997,305,542	0,002,692,982	1,00212E−06	4,73804E−07
	z_i_(r)	0,999,510,958	1,180,812,645	1,677,782,821	1,252,451,347
20	n_i_(r)	60,340,250	324,988	248	119
	v_i_(r)	0,994,636,912	0,005,357,039	4,08798E−06	1,96157E−06
	P_i_(r)	0,995,212,548	0,004,782,787	3,16721E−06	1,49746E−06
	z_i_(r)	0,999,421,594	1,120,066,359	1,290,720,792	1,309,936,907
25	n_i_(r)	60,183,943	480,873	539	250
	v_i_(r)	0,992,060,378	0,007,926,617	8,88477E−06	4,12095E−06
	P_i_(r)	0,992,525,023	0,007,463,589	7,73245E−06	3,6559E−06
	z_i_(r)	0,999,531,855	1,062,038,225	1,149,024,631	1,127,206,212
50	n_i_(r)	58,931,099	1,724,359	6524	3623
	v_i_(r)	0,971,408,741	0,028,423,997	0,00,010,754	5,97208E−05
	P_i_(r)	0,970,280,622	0,029,537,165	0,000,123,719	5,84944E−05
	z_i_(r)	1,001,162,674	0,962,312,996	0,869,229,672	1,020,967,027
100	n_i_(r)	54,368,847	6,156,875	91,789	48,094
	v_i_(r)	0,896,205,469	0,101,488,727	0,001,513,032	0,000,792,772
	P_i_(r)	0,884,010,982	0,113,073,602	0,001,979,506	0,00,093,591
	z_i_(r)	1,013,794,498	0,897,545,712	0,764,348,198	0,847,060,243

**Figure 6:**
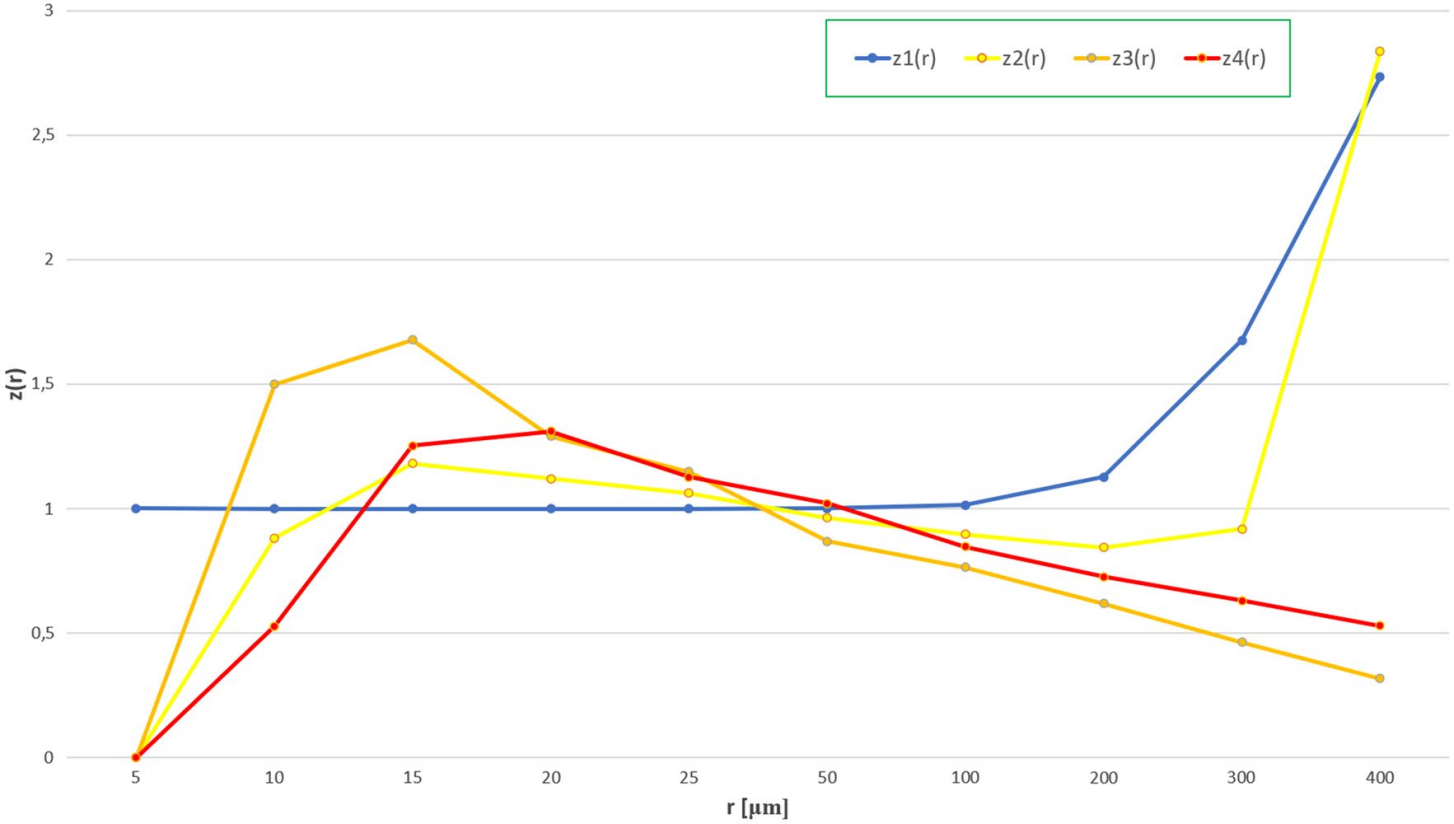
Analysis of grain distribution on the surface in relation to the random distribution. The grain sizes are marked on the horizontal axis, and the population concentration indicators for four different types are marked on the vertical axis.

The highest values of the indicators are achieved for the following range 15 μm < r < 20 μm. The ratio of the individual indicators is 1.18:1.68:1.31. For these values, it can be concluded that there is a slight concentration of grains, especially in linear systems that have values 1.68 times greater than the statistical random distribution. For values above 50 μm, all indices are below 1.0 and indicate compliance with the random distribution (Fig. 6).

**Figure 7:**
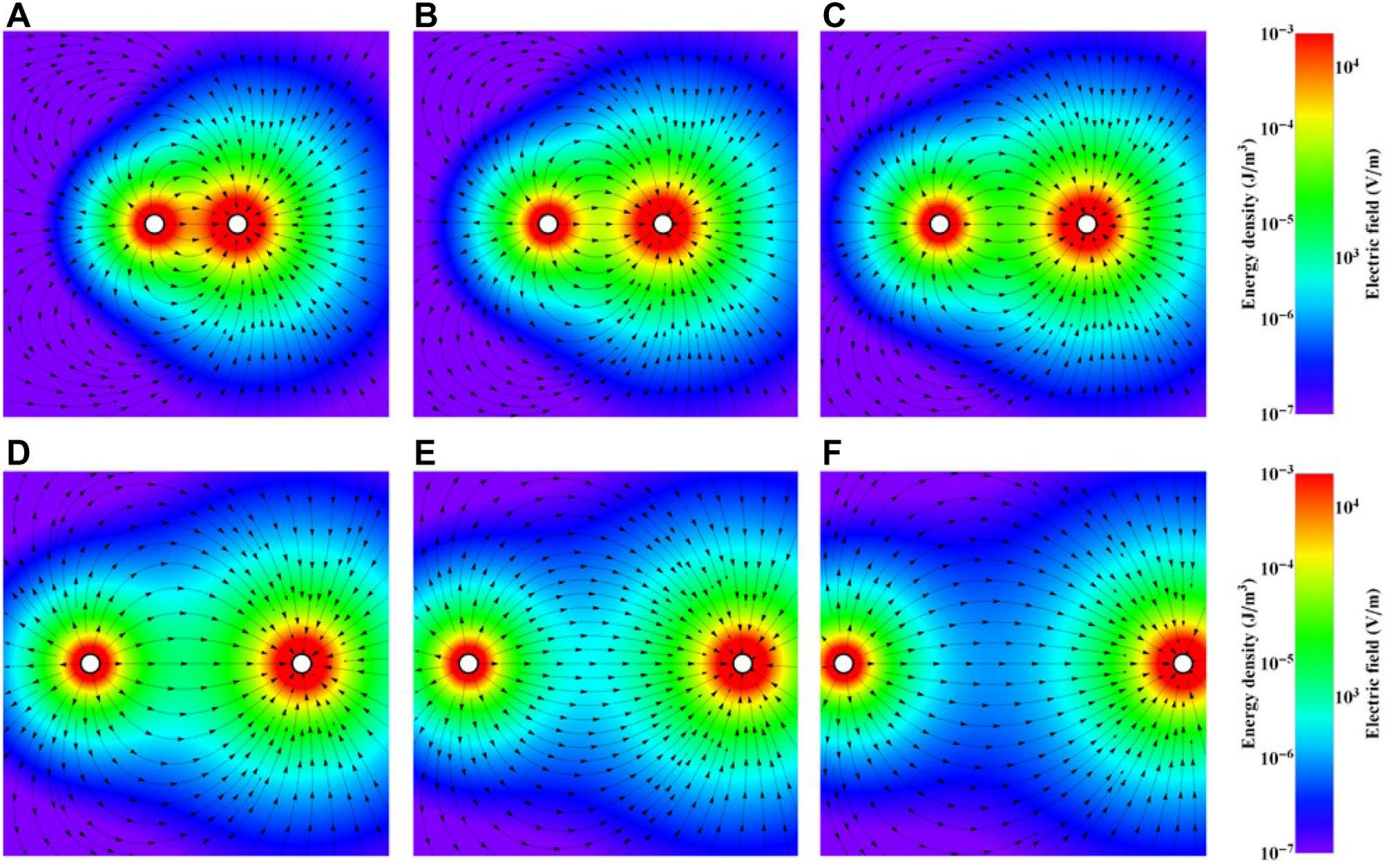
Modelling the electric field between electrodes at distances of (A) 50 µm, (B) 75 µm, (C) 100 µm, (D) 150 µm, (E) 200 µm, and (F) 250 µm. The colours show the energy density and electric field.

However, the maximum values of the indicators are relatively low and are not sufficient to state that the concentrations are massive and disturb the homogeneity and quality of the grain distribution. To assess the “electricidal effect” of the current between the zinc and copper electrodes, the finite element model (FEM) was used (Fig. 7). The electric field generated by the zinc anode and copper cathode was presented by using the finite element model under idealized vacuum conditions and with spherical electrodes that had the same diameters and same distances between them (see the Methods). Increasing the distance between electrodes caused the electric field to change from circles to more deformed ellipses that eventually divided into two disconnected ovals. The presented configuration and distance of galvanic microcells shows fundamental importance in regard to surface protection against viruses. We hypothesize that due to electrochemical processes, active biocidal Cu2 + and Zn2 + ions go directly to the vicinity of harmful microorganisms, and the electromagnetic field potential gradient facilitates the inactivation of viruses.

**Figure 8:**
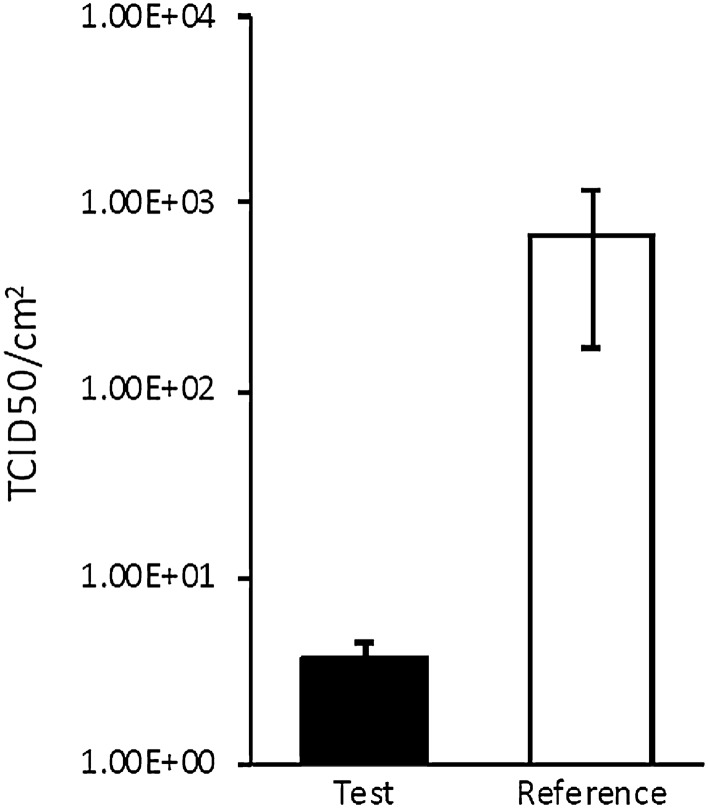
Mean TDIC50/cm^2^ values for human coronavirus NL63 following a contact time of 4 h with the test and reference control materials. Error bars represent the standard error of the mean. Source: Virology Research Service^20^.

The virucidal activity of galvanic microcells of zinc and copper contained within painted surfaces was evaluated using ISO 21702 (see the Methods). The treated material displays virucidal activity against human coronavirus NL63 with a contact time of 4 h (Fig. 8). The surface with galvanic microcells displays antiviral activity against human coronavirus NL63 (Tables 2, 3). The average recovered titre for the treated material was 3.73E + 00 TCID50/cm^2^ compared to the average recovered titre of 6.72E + 02 TCID50/cm^2^ for the nontreated reference control R (antiviral activity) = 2.26^20^.

**Table 2:** Average infectious units per cm^2^ recovered from the test and reference control materials after a contact time of 4 h with the virus. Source: Virology Research Service^20^.

Test condition	Virus recovery control (TCID50/cm^2^)	Antiviral test (TCID50/cm^2^)
Test	NA	3.73E + 00 ± 7.70E−01
Reference	9.67E + 04 ± 3.96E + 04	6.72E + 02 ± 5.03E+02

**Table 3:** Average infectious units per cm^2^ recovered from the test and reference control materials after a contact time of 4 h with the virus. Source: Virology Research Service^20^.

Test condition	TCID50 (log10)	R value	% Reduction
Test	0.57	2.26	99%
Reference	2.83		

Our results provide evidence that paint layers containing galvanic microcells with a total copper surface area of 4.4% display virucidal activity according to ISO 21702 standard with human coronavirus NL63 as a model organism. The average recovered titre for the treated material was 3.73E + 00 TCID50/cm^2^ compared to the average recovered titre of 6.72E + 02 TCID50/cm^2^ for the non-treated reference control. R (antiviral activity) = 2.26.

## References

[1] Basu S, Kabi P, Chaudhuri S, Saha A (2020). Insights on drying and precipitation dynamics of respiratory droplets from the perspective of COVID-19. Phys. Fluids.

[2] van Doramalen N (2020). Aerosol and surface stability of SARS-CoV-2 as compared with SARS-CoV-1N. Engl. J. Med..

[3] Ghafoor D, Khan Z, Khan A, Ualiyeva D, Zaman N (2021). xcessive use of disinfectants against COVID-19 posing a potential threat to living beings. Curr. Res. Toxicol..

[4] Vandenplas O, Wiszniewska M, Raulf M, de Blay F, Gerth van Wijk R, Moscato G (2014). EAACI position paper: Irritant-induced asthma. Allergy.

[5] Zock J-P, Vizcaya D, Le Moual N (2010). Update on asthma and cleaners. Curr. Opin. Allergy Clin. Immunol..

[6] Chen, T. A Rapid Review of Disinfectant Chemical Exposures and Health Effects During COVID-19 Pandemic. National Collaborating Centre for Environmental Health. ISBN: 978-1-988234-47-2 (2020).

[7] Decker, Franco lan. Volta and the 'Pile'. Electrochemistry Encyclopedia. Case Western Reserve University (2005).

[8] Prado JV, Vidal AR, Durán TC (2012). Aplicación de la capacidad bactericida del cobre en la práctica médica. Rev. Med. Chil..

[9] Sarah L, Warnes C, William K (2013). Inactivation of norovirus on dry copper alloy surfaces. PLoS ONE.

[10] Monk AB, Kanmukhla V, Trinder K (2014). Potent bactericidal efficacy of copper oxide impregnated non-porous solid surfaces. BMC Microbiol..

[11] Spisak W, Chlebicki A, Kaszczyszyn M (2016). Galvanic microcells as control agent of indoor microorganisms. Sci. Rep..

[12] EN 15457. Paints and varnishes - Laboratory method for testing the efficacy of film preservatives in a coating against fungi. Retrieved from https://www.en-standard.eu/csn-en-15457-paints-and-varnishes-laboratory-method-for-testing-the-efficacy-of-film-preservatives-in-a-coating-against-fungi/

[13] Polish Academy of Science. Report on the biocidal/biostatic properties of Galvi paint carried out in accordance with PN-EN 15457. Report No: 2/AL/2018, Cracow 2018.

[14] Spisak W, Chlebicki A, Kaszczyszyn M (2020). Three-electrode galvanic microcells as a new antimicrobial tool. Sci. Re.p.

[15] Gancarek Z, Idzik J, Spisak W (1991). A New method in Investigations of bubble cluster shapes in two—Phase flow. Chem. Eng. Process..

[16] Gancarek Z, Idzik J, Spisak W (1991). Three-circle method in the investigations of shapes of gas bubble clusters in two-phase flow. Chem. Eng. Process..

[17] Berry S, Giraldo NA, Green BF (2021). Analysis of multispectral imaging with the AstroPath platform informs efficacy of PD-1 blockade. Science.

[18] ISO 21702:2019 Measurement of antiviral activity on plastics and other non-porous surfaces. Retrieved from https://www.iso.org/standard/71365.html

[19] Tanabata T, Shibaya T, Hori K, Ebana K, Yano M (2021). SmartGrain: High-throughput phenotyping software for measuring seed shape through image analysis. Plant Physiol..

[20] Virology Research Service. Testing the virucidal activity of GALVI-19 against human coronavirus NL63 using ISO21702. Report No MS01, London, 1st March 2021.

